# Association between social isolation and reduced mental well-being in Swedish older adults during the first wave of the COVID-19 pandemic: the role of cardiometabolic diseases

**DOI:** 10.18632/aging.203956

**Published:** 2022-03-16

**Authors:** Abigail Dove, Jie Guo, Amaia Calderón-Larrañaga, Davide Liborio Vetrano, Laura Fratiglioni, Weili Xu

**Affiliations:** 1 Aging Research Center, Department of Neurobiology, Care Sciences and Society, Karolinska Institutet, Stockholm, Sweden; 2 Stockholm Gerontology Research Center, Stockholm, Sweden

**Keywords:** COVID-19, mental health, anxiety, depression, cardiometabolic disease

## Abstract

Social isolation has been recommended as a strategy for reducing COVID-19 risk, but it may have unintended consequences for mental well-being. We explored the relationship between social isolation and symptoms of depression and anxiety in older adults during the first wave of the COVID-19 pandemic and assessed the role of cardiometabolic diseases (CMDs) in this association. Between May and September 2020, 1,190 older adults from the Swedish National Study on Aging and Care in Kungsholmen were surveyed about their behaviors and health consequences during the first wave of the COVID-19 pandemic. In total, 913 (76.7%) participants reported socially isolating at home to avoid infection during this period. Social isolation was associated with a greater likelihood of reduced mental well-being (i.e., feelings of depression or anxiety) (OR: 1.74, 95% CI: 1.15-2.65). In joint exposure analysis, there was a significant likelihood of reduced mental well-being only among people who were socially isolating and had CMDs (OR: 2.13, 95% CI: 1.22-3.71) (reference: not isolating, CMD-free). In conclusion, social isolation as a COVID-19 prevention strategy was related to reduced mental well-being in an urban sample of Swedish older adults, especially among individuals with CMDs.

## INTRODUCTION

In early 2020, a highly infectious novel coronavirus (SARS-CoV2) began to spread across the world, leading the World Health Organization to declare COVID-19, the respiratory disease caused by the virus, an international public health emergency on January 30 and a global pandemic on March 11 [1]. Advanced age is a key risk factor for COVID-19 mortality [2]. Other factors associated with COVID-19 fatality include male sex and the presence of cardiometabolic comorbidities such as type 2 diabetes (T2D) and cardiovascular disease (CVD) [3, 4].

In an effort to curb the virus’ spread and reduce COVID-19 mortality, many countries implemented strict measures, including lockdowns and stay-at-home orders. In contrast, Sweden’s pandemic response emphasized voluntary adherence to recommendations from the Public Health Agency, including avoiding contact with others if one showed signs of COVID-19 symptoms, maintaining hand hygiene, and practicing social distancing [5]. Additionally, older adults in Sweden were further advised to stay at home and avoid crowded venues such as social gatherings and public transportation [5].

Though it is undoubtably effective for reducing the spread of infection, social isolation – that is, staying at home and minimizing in-person contact with others – may have unintended negative consequences. Before the COVID-19 pandemic, social isolation has been identified as a risk factor for poorer mental health [6, 7]. Consistent with this, recent studies have linked social isolation as a COVID-19 prevention measure with reduced mental health, both in the general adult population [8–10] and among older and more frail adults in particular [11–13].

Despite increased interest in the impact of pandemic-related social isolation, no studies to our knowledge have explored this issue in relation to the growing population of older adults with cardiometabolic disease. On one hand, people with cardiometabolic diseases (CMDs) stand to benefit most from social isolation from a COVID-19 prevention perspective, given the higher COVID-19 fatality rates associated with T2D and CVD [3]. On the other hand, other possible negative health consequences (e.g., reduced physical activity and cognitive decline) that accompany social isolation could be particularly damaging in this already-vulnerable population.

Pandemics are predicted to become more intense and more frequent with continued globalization and expansion. To this end, a recent investigation estimated that the probability of a COVID-19-scale pandemic is as high as 2% in any given year [14]. For the remainder of the current pandemic as well as for inevitable future ones, it is important to assess what is lost and gained by social isolation, particularly for vulnerable populations.

In this study, using data from the Swedish National Study on Aging and Care in Kungsholmen (SNAC-K), we aimed to assess the association of social isolation with depression and anxiety during the COVID-19 pandemic, and to examine the role of CMDs in this association. We hypothesized that social isolation would have an adverse impact on mental health and that these associations may be modified by the presence of CMDs.

## RESULTS

### Characteristics of the study population

Of 1,190 study participants (64.0% female, mean age 78.6 ± 8.2 years), 913 (76.7%) had been socially isolating during the first wave of the COVID-19 pandemic. Compared to those who did not isolate, socially isolating participants were more likely to be older, have a lower education level, and have CMDs, but were less likely to smoke or drink heavily (Table 1).

**Table 1:** Baseline characteristics of the study population (n = 1190).

**Variables**	**Total**	**Social isolation status**		
		**Not isolating (n=277)**	**Isolating (n=913)**	**P value**
Age (years)	78.6±8.2	74.4±7.2	79.9±8.1	<0.001
<80 years	623 (52.4)	205 (74.0)	418 (45.8)	<0.001
≥80 years	567 (47.7)	72 (26.0)	595 (54.2)	
Gender				0.192
Men	429 (36.0)	109 (39.4)	320 (35.1)	
Women	761 (64.0)	168 (60.7)	593 (65.0)	
Education				0.031
Elementary	41 (3.5)	4 (1.4)	37 (4.1)	
High school	460 (38.7)	98 (35.4)	362 (39.7)	
University	698 (57.9)	175 (63.2)	514 (56.3)	
Living alone	589 (49.6)	134 (48.4)	455 (50.0)	0.647
Smoking				<0.001
Never	826 (90.7)	190 (82.6)	636 (93.4)	
Former smoker	22 (2.4)	13 (5.7)	9 (1.3)	
Current smoker	63 6.9)	27 (11.7)	36 (5.3)	
Alcohol consumption				<0.001
No or occasional	192 (21.9)	27 (12.2)	165 (25.3)	
Light to moderate	507 (57.9)	140 (63.1)	367 (56.2)	
Heavy	176 (20.1)	55 (24.8)	121 (18.5)	
Pre-pandemic depressive symptoms score	2.0±2.7	2.0±2.6	2.5±3.2	0.0639
Cardiometabolic diseases	277 (23.3)	43 (15.5)	234 (25.6)	<0.001
Reduced mental health during the first wave of the COVID-19 pandemic	290 (24.4)	55 (19.9)	235 (25.7)	0.046
Depression	110 (9.2)	19 (6.9)	91 (9.10)	0.118
Anxiety	245 (20.6)	44 (15.9)	201 (22.0)	0.027

Data are presented as means ± standard deviations or number (proportion %).

Missing variables: 2 were missing data on living conditions, 279 on smoking status, 315 on alcohol consumption, and 276 on depressive symptoms score.

### Associations between social isolation status and mental well-being

Participants who had been socially isolating showed a higher likelihood of reduced mental well-being (multi- adjusted OR [95% CI]: 1.74 [1.15 – 2.65]) compared to those who were not isolating, including a higher likelihood of both depressive (2.08 [1.10 – 3.94]) and anxiety (1.82 [1.16 – 2.84]) symptoms (Table 2).

**Table 2:** Relationship between social isolation and mental health among older adults during the first wave of the COVID-19 pandemic.

	***n***	**Reduced mental well-being**		**Components of reduced mental well-being**			
				**Depressive symptoms**		**Anxiety symptoms**	
		OR (95% CI)*	***P*-value**	OR (95% CI)*	***P*-value**	OR (95% CI)*	***P*-value**
Social isolation							
No	277	Reference		Reference		Reference	
Yes	913	1.74 (1.15 – 2.65)	0.009	2.08 (1.10 – 3.94)	0.024	1.82 (1.16 – 2.84)	0.009

*Logistic regression model adjusted for baseline age, gender, education, living status, smoking status, alcohol consumption, and pre-pandemic depressive symptoms.

### Joint effect of social isolation status and CMDs on mental well-being

**Figure 1:**
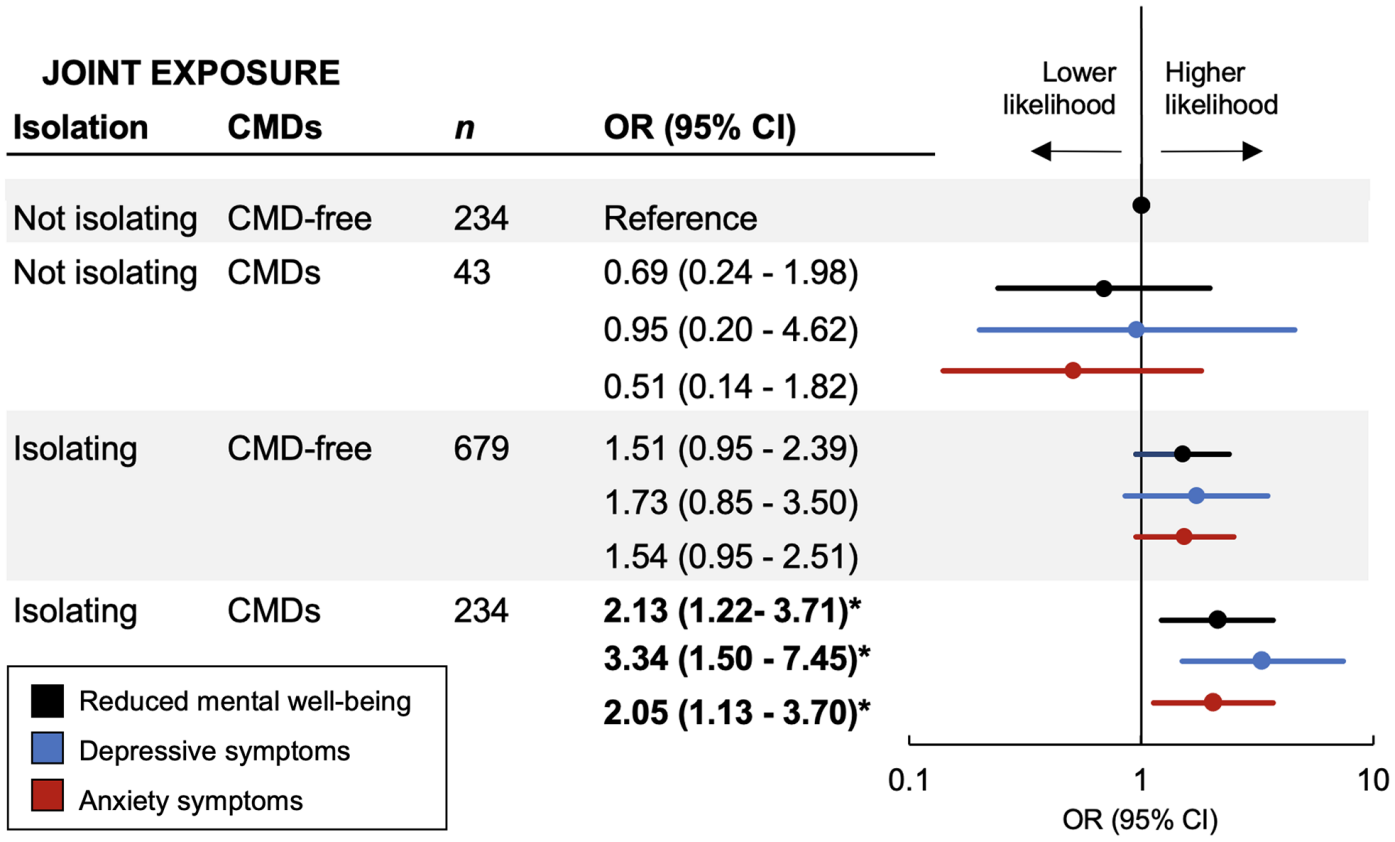
**Joint effect of social isolation and cardiometabolic diseases (CMDs) on mental well-being during the first wave of the COVID-19 pandemic.** Odds ratios (95% CIs) of reduced mental well-being, depressive symptoms, and anxiety symptoms from logistic regression models adjusted for baseline age, gender, education, living status, smoking status, alcohol consumption, and pre-pandemic depressive symptoms. Interaction between social isolation status and CMD status on reduced mental well-being: *P* for multiplicative interaction = 0.208; *P* for additive interaction = 0.02.

The presence of CMDs was not significantly associated with reduced mental well-being (OR 1.30, 95% CI 0.88 – 1.93) compared to those without CMDs. However, in joint exposure analysis, compared to CMD-free participants who were not socially isolating, participants who were socially isolating and had CMDs had over twice the likelihood of reduced mental well-being (OR 2,13, 95% CI 1.22 – 3.71). Similarly, there was a significantly increased likelihood (OR [95% CI]) of depressive (3.34 [1.50 – 7.45]) and anxiety (2.05 [1.13 – 3.70]) symptoms only among participants who both had CMDs and were socially isolating (Figure 1). There was a significant additive (p=0.02) but not multiplicative (p=0.208) interaction between social isolation status and CMDs on the likelihood of reduced mental well-being.

### Gender-specific effects of social isolation and CMDs on mental well-being

After stratifying by gender, the association between self-isolation and reduced mental well-being (OR, 95% CI) was statistically significant among women (1.80, 1.09-2.96). Moreover, in joint exposure analysis, the OR (95% CI) of reduced mental well-being was 2.26 (1.16-4.42) among women who were isolating and had CMDs compared to those who were CMD-free and not isolating. Among men, these associations had a similar effect size, but were not statistically significant (Table 3). There were no significant interactions, additive or multiplicative, between social isolation status and CMDs for men and women separately.

**Table 3:** Relationship between social isolation and mental well-being among older adults during the first wave of the COVID-19 pandemic, stratified by gender.

		**Men**				**Women**		
		***n***	OR (95% CI)*	***P*-value**		***n***	OR (95% CI)*	***P*-value**
**Social isolation**								
No		109	Reference			168	Reference	
Yes		320	1.84 (0.82 – 4.16)	0.139		593	1.80 (1.09 – 2.96)	0.021
**Joint exposure†**								
**Isolation**	**CMDs**							
No	No	85	Reference			149	Reference	
No	Yes	24	0.44 (0.08 – 2.58)	0.364		19	0.75 (0.19 – 2.96)	0.683
Yes	No	213	1.39 (0.54 – 3.55)	0.492		466	1.62 (0.95 – 2.77)	0.077
Yes	Yes	107	1.74 (0.61 – 4.95)	0.302		127	2.26 (1.16 – 4.42)	0.017

*Logistic regression models adjusted for baseline age, education, living status, smoking status, alcohol consumption, and pre-pandemic depressive symptoms.

^†^*P* for multiplicative interaction: 0.280 for men, 0.400 for women; *P* for additive interaction: 0.38 for men, 0.09 for women.

## DISCUSSION

In this population-based cohort study of older adults in Stockholm during the first wave of the COVID-19 pandemic, we found that: 1) social isolation was associated with higher levels of depression and anxiety and 2) reduced mental well-being was greatest among participants who were socially isolating and had CMDs.

Social isolation is a widely recognized risk factor for depression and anxiety and its effect on mental health may be particularly pronounced among older adults [6, 7]. The consequences of social isolation are particularly important to consider in the context of the COVID-19 pandemic, which forced people around the world into isolation at home on an unprecedented scale. While the full extent of the pandemic’s impact on mental health is still coming into view, studies conducted in the midst of the COVID-19 outbreak indicate that engaging in social isolation to avoid infection comes with significant collateral damage. The COVID-19 Mental Disorders Collaboration reported that reduced human mobility during the pandemic (calculated using mobile phone user data and information on physical distancing mandates) was associated with an increase in the prevalence of major depressive disorder and anxiety disorders globally [10]. A study including nearly 10,000 participants across 78 countries uncovered high levels of reduced mental health during the pandemic, but this was buffered by leaving home more often [9]. Another study reported a correlation between depressive symptoms and more days stayed at home during the pandemic [8]. Finally, a comprehensive review on the mental and physical effects of the pandemic on older people described consistent negative impacts of social distancing and social isolation on mental well-being – in particular, depression, anxiety, and reduced sleep quality [12].

Consistent with these reports, we found that social isolation was related to reduced mental well-being in the form of feelings of both depression and anxiety in an urban population of Swedish older adults. It is notable that these adverse effects of social isolation were apparent even in the context of Sweden’s comparatively relaxed COVID-19 prevention measures, where people who isolated themselves at home did so by individual choice and not because of an external mandate. Additional studies are needed to better understand the long-term impacts of COVID-19-related social isolation beyond the pandemic’s first wave.

Our study takes the further step of examining the impact of cardiometabolic disease on the association between social isolation and mental well-being. Older adults with CMDs like T2D and CVD have far greater risk of COVID-19 fatality [3] and were especially strongly encouraged to socially isolate early in the pandemic to avoid coronavirus infection. Furthermore, both T2D and CVD have been bidirectionally related to poor mental health [15], so people with CMDs may experience the negative impacts of social isolation especially acutely. Moreover, these individuals may be particularly worried about contracting COVID-19 given their pre-existing health conditions. However, to our knowledge, no previous study has explored mental well-being in relation to social isolation among the older adults with CMDs. We found that people with CMDs were particularly vulnerable to the negative mental health impacts of social isolation. In comparison to people who were CMD-free and not isolating, people with CMDs who isolated showed a 3-fold greater odds ratio for depressive symptoms and 2-fold greater odds ratio for anxiety symptoms.

The health consequences of social isolation for people with CMDs may be even more severe if social isolation itself impacts individuals’ ability to self-manage CMDs. Social isolation has been identified as a risk factor for coronary heart disease and stroke [16]. Furthermore, several studies have linked social isolation during the pandemic to reduced levels of physical activity among older adults [17–20], but additional research is needed to determine whether this translates to a worsening of glycemic control or the severity of cardiovascular disease.

A previous investigation indicated that loneliness, anxiety, and insomnia during the pandemic were particularly pronounced among women aged ≥60 years [11]. Consistent with this, our results indicate that the association between social isolation, CMDs, and reduced mental well-being is significant among women but not men. However, this might be due to a smaller sample size of men rather than a gender-specific effect of social isolation. Therefore, the potential greater susceptibility of women to the negative impacts of social isolation on mental well-being warrants further investigation in large population-based studies.

It is possible that the association between social isolation and reduced mental well-being was impacted by acute or time-limited anxiety deriving directly from the pandemic situation. We addressed this in sensitivity analyses stratified by participants’ self-reported levels of worry that they themselves (Supplementary Table 1) or a member of their family (Supplementary Table 2) would be affected by COVID-19. In both analyses, the association between social isolation and reduced mental well-being was numerically lower among participants with lower as opposed to higher levels of worry, though neither the less-worried nor the more-worried participants showed a statistically significant association between social isolation and reduced mental well-being, perhaps owing to the small sample size after stratification. By contrast, in a third sensitivity analysis stratified by participants’ self-reported feelings of nervousness and stress during the pandemic, social isolation was associated with twice the likelihood (OR, 95% CI) of reduced mental well-being among individuals with low levels of nervousness and stress (2.14, 1.10-4.15), but not those with high levels of nervousness and stress (0.97, 0.48-1.96). Supplementary Table 3 together these sensitivity analyses indicate that acute psychological reactions to the pandemic may have impacted our findings, but their role is complex and difficult to understand in the absence of longitudinal data on mental well-being over the course of the pandemic.

Strengths of this study include the use of a study sample from a well-characterized population-based study, SNAC-K, and the use of a questionnaire developed by a multidisciplinary team of experts. However, some limitations should be acknowledged. First, these findings are based on self-reported information and therefore may be affected by recall bias. Second, given the pandemic restrictions, participants’ mental well-being was measured in terms of self-reported feelings of anxiety and depression via a telephone interview, rather than a formal diagnosis of anxiety or depression based on a clinician’s visit. Third, in this study, data on mental well-being was not collected in the same way before and after the onset of the COVID-19 pandemic, thus we could only examine the cross-sectional relationship between social isolation and feelings of depression and anxiety. However, considering the possible influence of pre-pandemic mental health on mental well-being during the first wave of COVID-19, we included participants’ MADRS scores from the most recent regular SNAC-K follow-up visit (conducted from 2016 to 2019). Further population-based longitudinal studies are warranted to confirm the social isolation-mental wellbeing association. Additionally, the generalizability of these findings – which reflect an affluent, highly educated, urban population – may be limited, particularly given the voluntary nature of Sweden’s COVID-19 prevention recommendations, which differed substantially from the mandates issued in most other western countries. Finally, we could not rule out the influence of potential residual confounding due to unmeasured factors, like the number of social contacts or the severity of CMDs, which may be related to both mental health and the choice to self-isolate.

To the best of our knowledge, this is the first study to examine the combined impact of social isolation and CMDs on mental well-being during the COVID-19 pandemic. Pandemic-related social isolation was associated with depressive and anxiety symptoms in older adults, particularly those with cardiometabolic comorbidities. Our findings highlight the collateral damage of social isolation as a COVID-19 prevention strategy and underscore the need for mental health support for older adults during subsequent waves of the current pandemic. Furthermore, in the unfortunate but highly probable event of a future pandemic on the scale of COVID-19 [14], the amplified effects of social isolation on people with CMDs should be kept in mind and addressed earlier with focused preventive strategies. Together with reports from other geographical settings, the findings from our study may contribute to an open, cautious, and impartial discussion about what countries have gained and lost as a consequence of the exceptional public health measures (both mandated and voluntary) related to the COVID-19 pandemic.

## MATERIALS AND METHODS

### Study population

The study population was derived from SNAC-K, an ongoing population-based cohort study that includes a random sample of older adults aged ≥60 years living in central Stockholm, Sweden. Between May and September 2020, individuals who participated in the regular SNAC-K follow-up assessment in 2016-2019 were invited to participate in a structured survey administered over the telephone by trained SNAC-K staff to assess their behaviors and direct and indirect health consequences during the first wave of the COVID-19 pandemic (i.e., since March 2020). The telephone questionnaire was developed by the SNAC-K data collection team with input from experts in geriatric medicine, mental health, neurology, and public health. The questions contained some items from the regular SNAC-K assessments (which participants undergo every 3 or 6 years) as well as items from the WHO Europe survey tool [21]. Before the interview, SNAC-K staff explained to participants that all questions referred specifically to the pandemic context. People with a known diagnosis of dementia, very impaired hearing, and those who were living in care or nursing homes were excluded from the telephone questionnaire. Of 1,231 participants who completed the telephone questionnaire (91.9% response rate), we excluded 25 with missing information on pandemic-related mental well-being (i.e., depressive and anxiety symptoms) and 16 with missing information on social isolation, leaving a total of 1,190 participants for the current study (Supplementary Figure 1).

The study was approved by the Karolinska Institutet Ethical Committee and the Regional Ethical Review Board in Stockholm, Sweden. All participants provided informed and written consent.

### Data collection

The SNAC-K protocol has been described in detail previously [22]. Briefly, at each wave of follow-up, trained nurses and physicians collected data on demographic factors, lifestyle factors, medication use, and medical history. Additionally, blood samples were collected for laboratory tests (e.g., glycated hemoglobin).

Education level was defined as elementary, high school, or university. Smoking status was grouped into never, former, or current smokers. Alcohol consumption was categorized as no/occasional, light-to-moderate (1–14 drinks/week for men or 1–7 drinks/week for women), or heavy (>14 drinks/week for men or >7 drinks/week for women) drinking [23]. Living status was dichotomized as living alone or not living alone (including living with a partner, children or grandchildren, siblings or friends, etc.). Pre-pandemic levels of depressive symptoms were defined as participants’ Montgomery-Asberg Depression Rating Scale (MADRS) score during the most recent regular SNAC-K follow-up assessment (conducted from 2016 to 2019) [24, 25]. Information on medical history collected from the Swedish National Patient Registry (NPR) using codes from the International Classification of Disease, 10^th^ version (ICD-10).

### Assessment of social isolation

In the telephone questionnaire, participants were asked to report whether or not they had been staying at home and avoiding in-person social contact to minimize their chances of being infected by the coronavirus. Social isolation status was dichotomized as isolating vs. not isolating.

### Assessment of mental well-being

The telephone questionnaire included questions to assess mental well-being since the onset of the COVID-19 pandemic in Sweden in March 2020. Participants’ experience of depression during the pandemic was assessed by the question, “Have you experienced depressive symptoms since March 2020?” Participants responded using a 1 to 6 scale, with 1 indicating a “neutral mood” and 6 indicating “consistent experience of maximum depression.” Participants were coded as having experienced depressive symptoms if they scored 3 (i.e., “predominant experience of depression, but brighter moments occur”) or higher. Participants’ experience of anxiety during the pandemic was ascertained by two questions: 1) “Have you experienced feelings of anxiety since March 2020?” (ranging from 1, “mostly calm,” to 6, “prolonged panic attacks; overwhelming feelings of fear that cannot be overcome on their own”); and “Have you experienced anxiety symptoms since March 2020?” (ranging from 1, “no excessive anxiety,” to 6, “disabling anxiety; constant brooding over small things, calming assurances have no effect”). Participants were coded as having experienced anxiety if they scored 3 or higher on either question. We additionally created a combined mental well-being endpoint defined as reduced mental well-being (i.e., experience of either depression or anxiety) or sound mental well-being (i.e., experience of neither depression nor anxiety).

### Assessment of CMDs

CMD status, defined as the presence of T2D and/or CVD [26], was assessed at the latest regular SNAC-K follow-up visit using data from multiple sources. T2D was ascertained based on self-reported medical history, glucose-lowering medication use, medical records from the NPR (ICD-10 code E11), or glycated hemoglobin ≥6.5% [27]. CVD was identified based on self-reported medical history or medical records from the NPR (including ischemic heart disease [ICD-10 codes: I20-22, I24-25, Z951, and Z955], atrial fibrillation [code I48], heart failure [I110, I130, I132, I27, I280, I42–43, I50, I515, I517, I528, Z941, and Z943], cerebrovascular disease [G45-46, I60-64, I67, and I69], or other cardiovascular diseases [I09, I281, I310-311, I456, I495, I498, I70-72, I790-791, I950-951, I958, Q20-21, Q24-28, and Z958-959]).

### Statistical analyses

Characteristics of socially isolating vs. not isolating participants were compared using Chi-square tests for categorical variables and t tests for continuous variables. Data were presented as numbers and percentages for categorical variables and as means and standard deviations for continuous variables.

Odds ratios and 95% confidence intervals for the association between social isolation status and depression, anxiety, or overall reduced mental well-being were obtained from logistic regression analyses. Models were adjusted for age, gender, education, living status, smoking status, alcohol consumption, and pre-pandemic levels of depressive symptoms (i.e. MADRS score from the most recent regular SNAC-K follow-up visit).

We additionally assessed the joint effect of isolation status and CMDs on mental well-being. Participants were categorized into four groups according to combined social isolation (yes vs. no) and CMD (CMD-free vs. any CMD) status. We assessed the additive interaction between social isolation status and CMDs using the attributable proportion due to interaction (AP). We examined the multiplicative interaction between social isolation status and CMDs by adding the cross-product term (social isolation status × CMD status) into the model. To assess possible gender-specific aspects of the associations between social isolation, CMDs, and mental well-being, we repeated all analyses separately among men and women.

Finally, we accounted for potential acute or time-limited anxiety deriving directly from the pandemic situation in sensitivity analyses stratified by participants’ level of worry about being affected by COVID-19 (“not at all,” “somewhat,” or “moderately” vs. “very” or “extremely”), level of worry about family members being affected by COVID-19 (“not at all,” “somewhat,” or “moderately” vs. “very” or “extremely”), and feelings of nervousness and stress (“never,” “rarely,” or “sometimes” vs. “quite often” or “very often”) since the beginning of the pandemic.

All *P*-values were two-sided, and *P*-values <0.05 were considered statistically significant. Statistical analyses were performed using Stata SE 16.0 (StataCorp, College Station, TX).

## Supplementary Material

Supplementary Figure 1

Supplementary Tables
