# Supplementary figures and images for: Association between social isolation and reduced mental well-being in Swedish older adults during the first wave of the COVID-19 pandemic: the role of cardiometabolic diseases
Source: Aging (Albany NY). 2022 Mar 16;14(6):2462–74. doi: 10.18632/aging.203956 (PMC9004574; doi:10.18632/aging.203956)

## SUPPLEMENTARY FIGURE

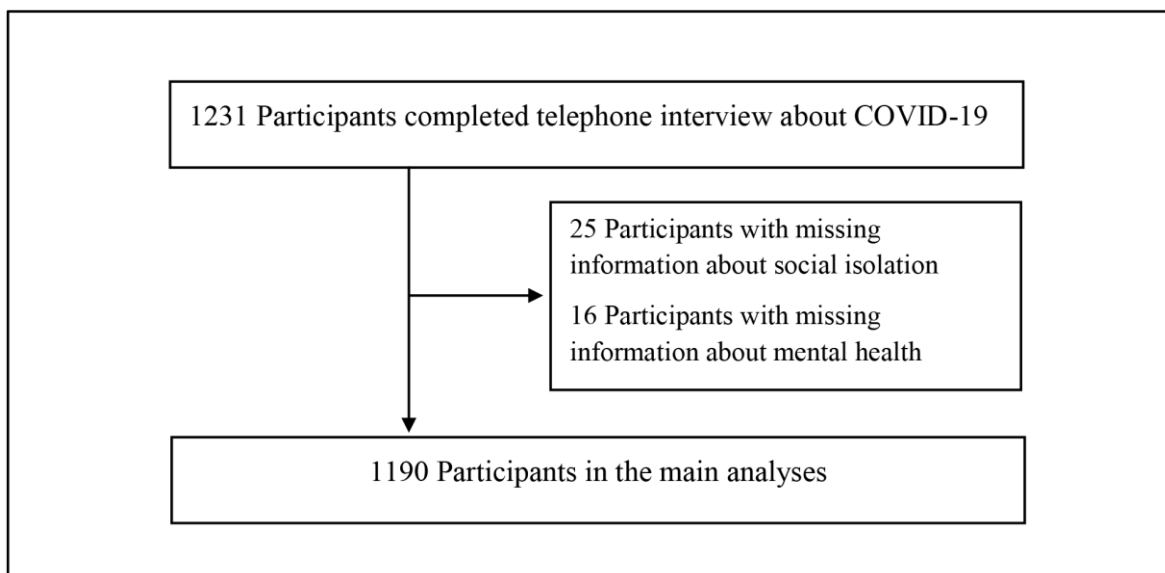

**Supplementary Figure 1. Flow chart of the study population.**

Supplement: Supplementary Figure 1 [file aging-14-203956-s001.pdf]
