# Supplementary material for: Association between social isolation and reduced mental well-being in Swedish older adults during the first wave of the COVID-19 pandemic: the role of cardiometabolic diseases
Source: Aging (Albany NY). 2022 Mar 16;14(6):2462–74. doi: 10.18632/aging.203956 (PMC9004574; doi:10.18632/aging.203956)
Supplement: Supplementary Tables [file aging-14-203956-s002.pdf]

SUPPLEMENTARY TABLES

Supplementary Table 1. Relationship between social isolation and mental well-being among older adults during the first wave of the COVID-19 pandemic, stratified by level of worry about being affected by COVID-19.

		Are you worried about being affected by COVID-19?			
		Not at all / Somewhat / Moderately		Very / Extremely	
		n	OR (95% CI)*	*n*	OR (95% CI)*
**Social isolation**					
No		254	Reference	20	Reference
Yes		750	1.33 (0.83 – 2.14)	153	1.65 (0.44 – 6.19)
**Joint exposure**					
**Isolation**	**CMDs**				
No	No	211	Reference	20	Reference
No	Yes	43	0.81 (0.28 – 2.35)	0	--
Yes	No	560	1.17 (0.70 – 1.98)	113	1.80 (0.47 – 6.90)
Yes	Yes	190	1.71 (0.89 – 3.27)	40	1.26 (0.28 – 5.80)

*Logistic regression models adjusted for baseline age, sex, education, living status, smoking status, alcohol consumption, and pre-pandemic depressive symptoms.

Supplementary Table 2. Relationship between social isolation and mental well-being among older adults during the first wave of the COVID-19 pandemic, stratified by level of worry about family members being affected by COVID-19.

		Are you worried that someone in your family will be affected by COVID-19?			
		Not at all / Somewhat / Moderately		Very / Extremely	
		n	OR (95% CI)*	*n*	OR (95% CI)*
**Social isolation**					
No		232	Reference	42	Reference
Yes		651	1.39 (0.84 – 2.31)	251	1.74 (0.73 – 4.13)
**Joint exposure**					
**Isolation**	**CMDs**				
No	No	196	Reference	36	Reference
No	Yes	36	0.83 (0.25 – 2.71)	6	0.30 (0.3 – 3.35)
Yes	No	478	1.21 (0.69 – 2.12)	193	1.36 (0.53 – 3.48)
Yes	Yes	173	1.81 (0.92 – 3.57)	58	1.86 (0.60 – 5.83)

*Logistic regression models adjusted for baseline age, sex, education, living status, smoking status, alcohol consumption, and pre-pandemic depressive symptoms.

Supplementary Table 3. Relationship between social isolation and mental well-being among older adults during the first wave of the COVID-19 pandemic, stratified by level of nervousness and stress during the first wave of the pandemic.

		How often have you felt nervous or stressed since March 2020?			
		Never / Rarely / Sometimes		Quite often / Very often	
		n	**OR (95% CI)***	***n***	**OR (95% CI)***
**Social isolation**					
No		216	Reference	59	Reference
Yes		648	**2.14 (1.10 – 4.15)**	226	0.97 (0.48 – 1.96)
**Joint exposure**					
**Isolation**	**CMDs**				
No	No	179	Reference	53	Reference
No	Yes	37	0.90 (0.18 – 4.40)	6	0.64 (0.10 – 4.07)
Yes	No	476	1.91 (0.92 – 3.99)	175	0.87 (0.41 – 1.84)
Yes	Yes	172	2.75 (1.16 – 6.51)	51	1.16 (0.43 – 3.15)

*Logistic regression models adjusted for baseline age, sex, education, living status, smoking status, alcohol consumption, and pre-pandemic depressive symptoms.
